# A Singular Case Analysis: Lamotrigine‐Associated Stevens–Johnson Syndrome

**DOI:** 10.1155/crcc/4835223

**Published:** 2024-11-26

**Authors:** Albin Joshi, Anjula Palikhe, Santosh Acharya, Puskar Kunwor

**Affiliations:** ^1^ Department of Pharmacy, KIST Medical College and Teaching Hospital, Imadol, Lalitpur, Nepal, kistmcth.edu.np; ^2^ Department of Critical Care, KIST Medical College and Teaching Hospital, Imadol, Lalitpur, Nepal, kistmcth.edu.np; ^3^ Department of Clinical Pharmacy, Nepal Cancer Hospital and Research Center, Harisiddhi, Lalitpur, Nepal

**Keywords:** adverse drug reaction (ADR), antiepileptic drugs, case report, lamotrigine, Stevens–Johnson syndrome (SJS), toxic epidermal necrolysis (TEN)

## Abstract

Stevens–Johnson syndrome and toxic epidermal necrolysis (SJS/TEN) is an immune complex–mediated hypersensitivity reaction linked as an adverse side effect to many drugs. There have been case reports of similar incidences in Nepal related to various medications. Here, we present a case of a 29‐year‐old lady who developed a generalized erythematous rash over her body and erosion of the oral mucous membrane. Two weeks back she gave a history of initiation of lamotrigine, olanzapine, and sertraline. Given the strong association between SJS and lamotrigine, and the usual presentation being within the first 8 weeks of exposure to susceptible medications; she was diagnosed as SJS/TEN induced by lamotrigine. On April 1, 2024, she was admitted to the ICU at KIST MCTH. All the medicines were withheld, and she was managed with corticosteroids and antihistamines. She improved significantly within 7 days. Early identification of SJS, discontinuation of triggering medicines, and prompt initiation of supportive therapy improved the prognosis.

## 1. Introduction

According to the World Health Organization (WHO), an adverse drug reaction (ADR) is a response to a drug that is noxious and unintended and which occurs at doses normally used in man for the prophylaxis, diagnosis, or therapy of disease or the modification of physiological functions [1]. In Nepal, the prevalence of ADRs was found to be 0.86%, the male‐to‐female ratio to be 0.85, and 10.81% of the ADRs were considered to be severe. Maximum numbers of ADRs reported were related to skin reactions, 35.13% [2]. ADRs are one of the leading causes of mortality among hospitalized patients. They may vary from mild rashes to severe reactions such as SJS [3].

Stevens–Johnson syndrome (SJS) is a form of life‐threatening skin condition, in which cell death causes the epidermis to separate from the dermis. SJS is a Type IV hypersensitivity reaction with the release of various cytotoxic signals activated by cytotoxic T lymphocytes and natural killer cells that involves the skin and mucous membrane, characterized by erythematous rash, erosions, and detachment of the epidermis, with an estimated incidence of 1.0–6.0 cases per million [4–6]. SJS and TEN are two forms of life‐threatening skin conditions. They are separated based on body surface area (BSA) with SJS having < 10% and toxic epidermal necrolysis having > 30% involvement [5]. The mortality from these conditions ranges from 5% to 40%; SJS and TEN are characterized by extensive necrosis and erosions of the skin and mucous membrane leading to severe pain and restricted mobility, eating, chewing, speaking, and swallowing. Complications may be due to secondary infection which can increase mortality [7].

The most common causes for initiation of the syndrome are associated with drugs. Common anticonvulsants associated with SJS are carbamazepine, phenytoin, phenobarbital, fosphenytoin, valproic acid, lamotrigine, and oxcarbazepine. Lamotrigine falls under the high‐risk category for causing SJS, but the list is not exhaustive, and case reports of various medicines have been reported [8].

Lamotrigine is an antiseizure/antiepilepsy drug considered as first‐line treatment for primary generalized tonic–clonic seizures; off‐label uses include treatment of rapid‐cycling bipolar depression and bipolar disorder Type I maintenance [9]. SJS frequently occurs within the first 8 weeks of therapy as a response to certain drugs, commonly antiepileptics (carbamazepine, phenobarbital, phenytoin, lamotrigine, and sodium valproate), antibiotics (sulfonamides, penicillin, macrolides, fluoroquinolone, and tetracycline), antitubercular drugs (ethambutol and rifampicin), nonsteroidal anti‐inflammatory drugs (acetaminophen and aspirin). Anticonvulsants such as lamotrigine fall under the high‐risk level as a causative medication for triggering SJS based on current evidence [8, 10].

## 2. Case Report

**Figure 1:**
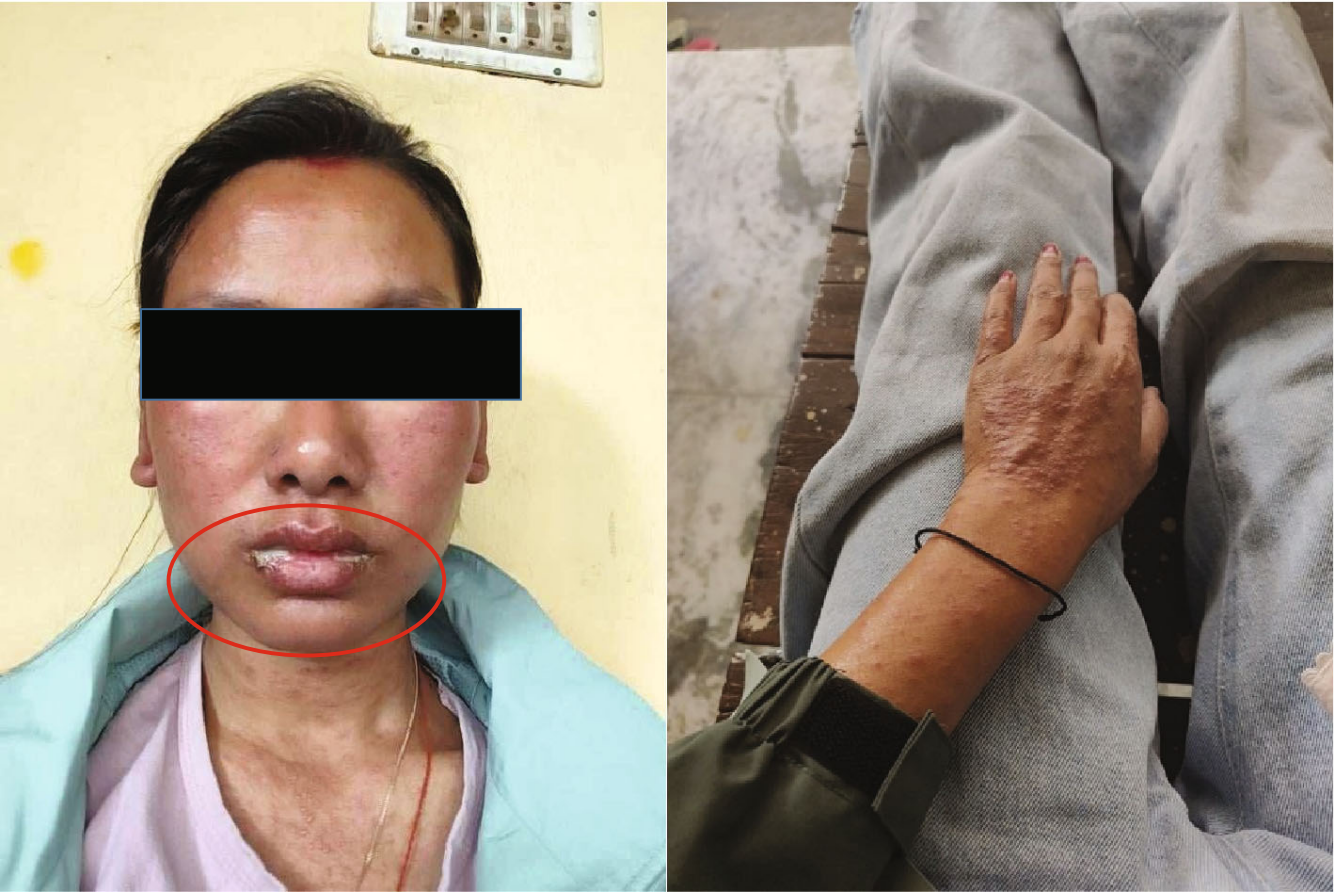
Initial presentation of oral mucosa erosion and maculopapular rashes.

**Figure 2:**
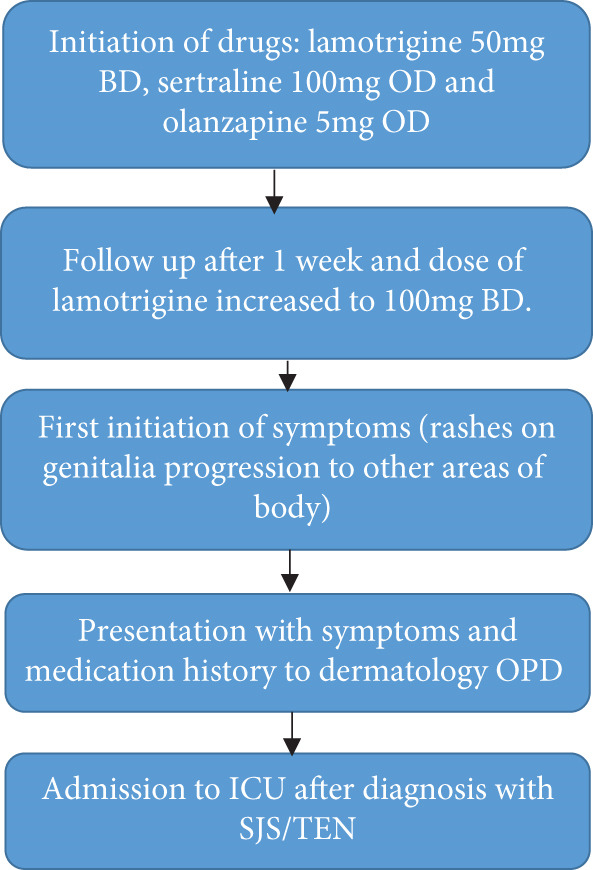
Flowchart of a sequence of events of the patient leading to SJS/TEN diagnosis.

A 29‐year‐old lady presented to the dermatology outpatient department (OPD) with a chief complaint of a maculopapular rash pruritic with painful raised bumps(Figure 1), spread throughout the body involving the upper trunk to her extremities, her face where mucous membrane detachment was present with plaque forming, causing difficulty in eating and swallowing, and itching of eyes for 7 days. Initially, she developed rashes on her upper trunk which slowly progressed to bilateral palms, soles, and extremities. Upon further questioning, she also had given symptoms of malaise, sore throat, and fever around the time the rash started to develop. Edema of the face was more noticeable around the eyes and lips on presentation as shown in Figure 1. After a review of her medication history, previous diagnosis, and the presentation of her symptoms, she was diagnosed with SJS/TEN and admitted to the intensive care unit (ICU) of our hospital for further management. According to her medical history, she was prescribed lamotrigine (50 mg) twice daily, olanzapine (5 mg) once daily, and sertraline (100 mg) once daily for recurrent depressive disorder by neuropsychiatry at our hospital on March 12, 2024. After 1 week on March 19, 2024, she returned for a follow‐up, and her dose of lamotrigine was increased to 100 mg BD. After 1 week of her dose being increased of lamotrigine, she started to develop the symptoms of lamotrigine‐induced SJS/TEN. Compared to British National Formulary (BNF) recommendations, the initial dose and dose titration for lamotrigine are higher in our case, that is, 100 mg once daily and increased to 200 mg twice daily in 7 days. The recommended initial dose for adults is 25 mg/day for the first 2 weeks, increased to 50 mg/day for a further 2 weeks with subsequent increases of up to 100 mg every 1–2 weeks until the required dose is reached. Maintenance doses are typically 100–200 mg/day in one or two divided doses and may be increased to up to 400 mg/day [11]. The sequence of events that led to admission is described in the flowchart (Figure 2).

On initial assessment, erosion of oral mucosa was present, and crusting was present over the bilateral angle of the mouth with the patient having symptoms of difficulty in swallowing, itching of eyes, and tenderness over genitalia and mouth. Nikolsky sign was negative, and around 10%–30% BSA was involved, so SJS/TEN overlap was present. Ophthalmology review suggested pseudomembranous conjunctivitis which was managed with fluorometholone 0.1%, carboxymethylcellulose 1% w/v, ofloxacin eye drops, and ciprofloxacin eye ointment with periodic pseudo membrane removal with wet swab. Severity‐of‐Illness Score for Toxic Epidermal Necrolysis (SCORTEN) for predicting the severity of SJS was used. In the SCORTEN scale, seven independent risk factors are systematically scored [12]. On admission, the patient is of age less than 40 (29 years female), with no associated cancer, Heart rate was below 120 (115 bpm), blood urea nitrogen was less than 28 mg/dL (21 mg/dL), detached/compromised surface area was greater than 10% (oral mucosa and ocular involvement), serum bicarbonate was greater than 20 mEq/L (23 mEq/L), and serum glucose was less than 250 mg/dL (114 mg/dL); based on these factors, the SCORTEN score was calculated to be 1 (mortality rate 3.2%). All her routine blood tests were normal except C reactive protein (CRP) (10.6 mg/dL). Internal organ involvement of the liver was only present as there were slight elevations of liver enzymes alanine aminotransferase (ALT) (45 U/L) and aspartate aminotransferase (AST) (50 U/L), but bilirubin values were within normal range, and renal function tests were normal for the patient. Tablet lamotrigine, olanzapine, and sertraline were all stopped on admission to the ICU. After a dermatology review, previous medications were advised to stop. Supportive measures were started on the patient with an injection of methylprednisolone (125 mg) intravenous (IV) for three doses along with an antihistamine tablet per oral (PO) cetirizine (10 mg) at bedtime and an injection of Ranitidine (50 mg) IV three times a day. Clinical pharmacists had advised the ICU team to start the patient on AVIL (pheniramine 22.75 mg) through the IV route four times a day and on a per‐need basis and calamine lotion on the affected BSAs due to persistent and severe itching complaints. After a literature review of the drug and case reports related to clonazepam/benzodiazepines induced SJS, the clinical pharmacist team suggested clonazepam as a safe option as ICU physicians wanted advice on whether clonazepam can be prescribed in such cases as sleep aid/antianxiolytic. Clinical pharmacists reviewed the patient’s ongoing medication and found no significant drug interactions. Calamine lotion was stopped as the patient complained of dryness of the skin due to it, which was then changed to clobetasol lotion and coconut oil applied topically. Oral lesions were treated with mupirocin ointment application and normal saline (NS) irrigation, chlorhexidine gargle, and triamcilone acetonide gel.

**Figure 3:**
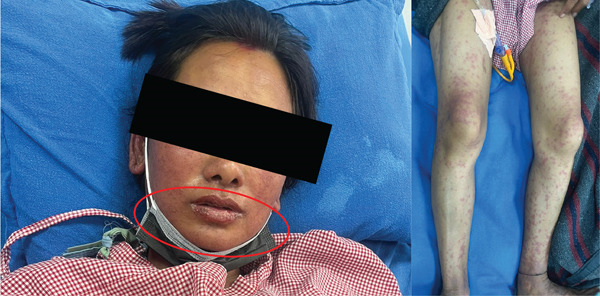
Oral mucosa erosion and rashes present during ICU stay.

**Figure 4:**
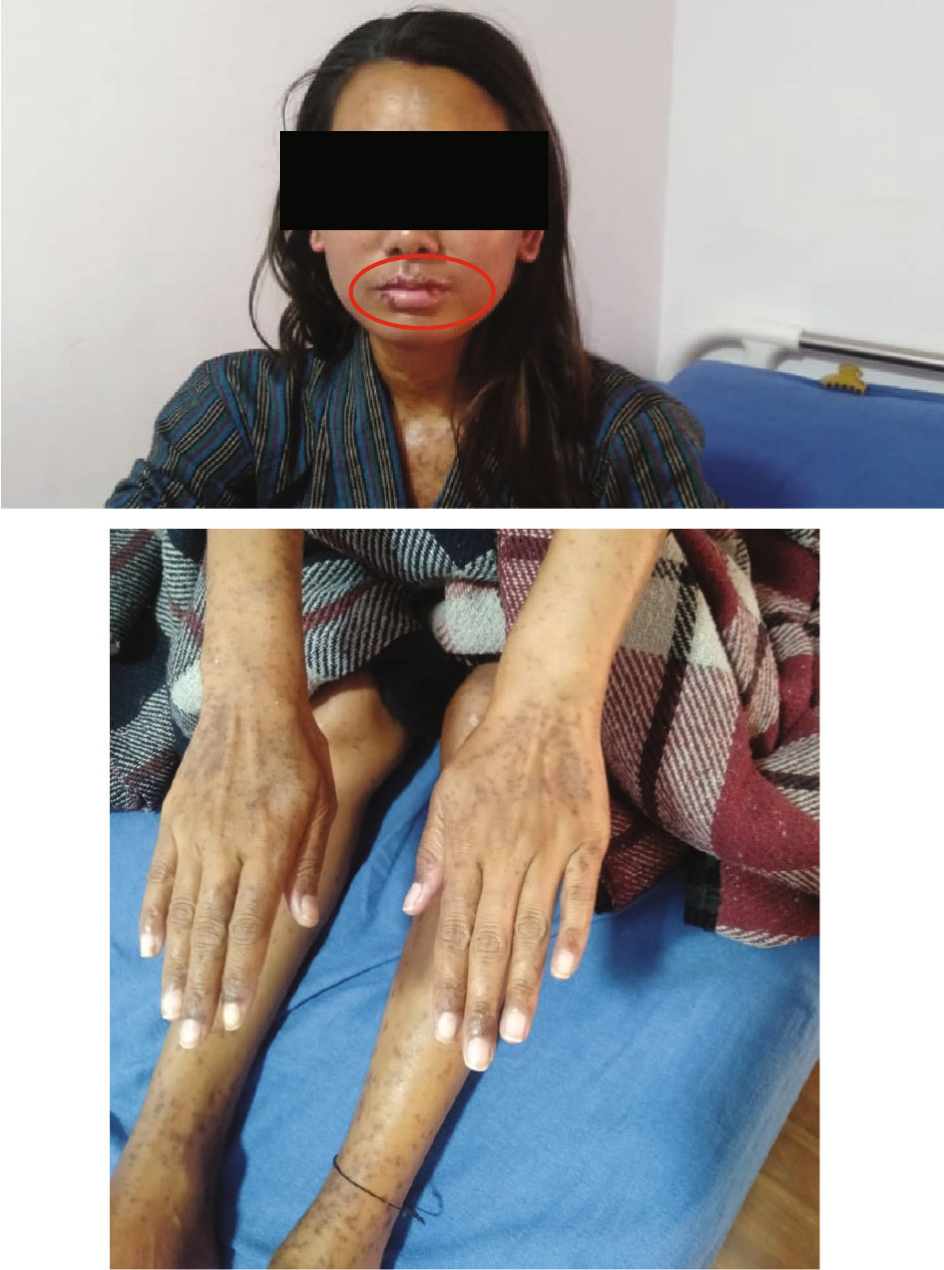
Resolving oral mucosa erosion and rashes during ward stay.

**Figure 5:**
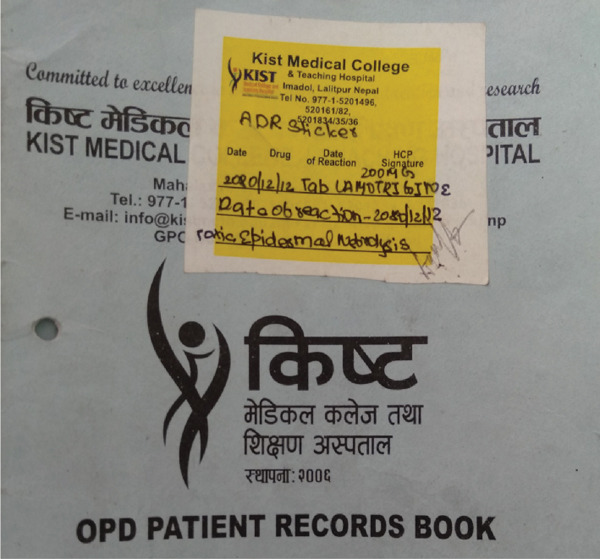
Adverse drug reaction alert sticker implementation in the patient document.

She stayed at the ICU for 4 days and was then shifted to the general ward as she was stable concerning symptoms of organ involvement due to SJS/TEN, as repeat liver function tests, renal function tests, and bloodwork came back normal. Rash and erosion of oral mucosa were slowly resolving through ICU stay (Figure 3) compared to the initial presentation. But, after a neuropsychiatric review, they diagnosed her with active suicidal tendencies. She was advised to stay in the psychiatry ward for further observation, and she was followed up by dermatology, ophthalmology, and clinical pharmacist at the psychiatry ward until her condition improved. At the time of her discharge from the hospital, her oral mucosa erosion had healed, the initial plaque formed had exfoliated, itching complaints had subsided significantly, and maculopapular rash scars were only remaining (Figure 4). The healing process for her affected mucosal membranes with wound care took 7 days, and her rashes were still present with some itching but managed with medications on discharge. On discharge, she was prescribed lithium carbonate (300 mg) twice daily and hydroxyzine (25 mg) at bedtime. Pharmacotherapy counseling regarding lithium was done regarding its adverse effects (seek immediate medical contact if severe nausea and vomiting, severe hand tremors, confusion, and vision changes occur). ADR alert sticker, recently developed at the hospital and designed by clinical pharmacists, was attached to patient hospital documents and on the outpatient card (Figure 5).

## 3. Discussion

SJS can present with fever, malaise, sore throat, cough, mucocutaneous lesions with blisters, and other systemic symptoms such as eye redness which may even result in visual impairment [13]. The initial nonspecific symptoms may mislead the diagnosis, delaying the treatment. The primary treatment comprises immediate identification and withdrawal of the offending drug followed by medication and supportive care [14].

SCORTEN scale is used as a predictor of the mortality rate in SJS/TEN patients. It is calculated by factors such as the age of the patient, tachycardia, total BSA involved, increased serum urea, serum glucose, and bicarbonate levels. A score of 5 or more indicates a mortality rate of 90% [15].

In our case, the patient developed SJS symptoms of maculopapular pruritic rashes and flu‐like symptoms (sore throat, malaise, and fever) a week after increasing the dose of lamotrigine. Severe reactions like SJS are more likely when lamotrigine is administered at an elevated starting dose or rapidly titrated, and slow titration of lamotrigine dose is recommended to decrease the risk for incidence of rashes [14, 16]. This is one of the risk factors for the development of SJS and a possible drug interaction between the medications she was taking alongside lamotrigine, that is, sertraline and olanzapine [17]. After reviewing the literature on drug interaction, we found that there is not a significant interaction between lamotrigine and olanzapine. However, with sertraline, there may be a possibility that lamotrigine drug levels were increased by decreasing its metabolism [18, 19]. Based on the review of existing case reports and literature, higher incidences were found among antiepileptics in our case of lamotrigine association with SJS/TEN syndrome in comparison to antipsychotics and selective serotonin reuptake inhibitors (SSRIs), where few case reports have been published. As lamotrigine has a stronger association with SJS/TEN and symptoms occurred after the dose of lamotrigine was increased, we did not suspect olanzapine and sertraline as the causative agent [20–22].

Medical treatment in our case included removal of the suspected offending agents, IV and oral corticosteroids, antihistamines, topical lotions for rashes, and proper wound care. Even though SJS is classified as a Type 4 hypersensitivity reaction that is cell mediated, due to the pruritic nature of the rash, it was not resolving even after IV corticosteroid use; antihistamines were chosen, which resolved the itching complaints of the patient [23].

The role of clinical pharmacists in ADR management is still not well understood in the Nepalese context, as pharmacists are generally involved only in ADR reporting and documentation [24]. Clinical pharmacists play an important role in identifying ADRs through clinical judgment, monitoring of patients, and their knowledge of pharmacology. They help in detecting such events and can alert physicians or suggest possible ways to manage such unwanted events related to pharmacotherapy [25]. In our center, the involvement of pharmacists in the detection and management of ADR is a new practice where pharmacists work in collaboration with the ICU team.

The key takeaway from our case report is that clinical pharmacists’ support plays an important role in managing such adverse reactions in collaboration with other healthcare professionals.

## 4. Conclusion

SJS is associated with several medications and is one of the dreadful ADR with a high incidence of mortality. So, taking a proper medication history of patients and identifying the initial nonspecific symptoms correlating to medications taken and when they started developing after taking medications can help identify the condition as quickly as possible for identification of causative agent and withdrawal of the drug. Pharmacists and prescribers should be aware of such incidences related to medications and counsel patients on the risk of developing SJS based on the medications they are receiving and what conditions they should seek for immediate medical attention. All healthcare professionals play a role in early diagnosis and treatment/supportive care of such conditions to prevent further harm and decrease mortality among patients diagnosed with SJS/TEN.

## Consent

The authors confirm that the patient has provided written informed consent.

## Conflicts of Interest

The authors declare no conflicts of interest.

## Author Contributions

P.K. and S.A. were involved in the conceptualization of the case report. A.J. was responsible for the literature review and writing the original draft. A.J. and P.K. obtained the consent from patient relatives. P.K., A.J., A.P., and S.A. were responsible for the review and editing of the prepared manuscript. P.K. was responsible for the supervision. P.K. and S.A. approved the final version. All authors accepted the final manuscript for publication.

## Funding

No funding was received for this research.

## Data Availability

The data that support the findings of this study are available on request from the corresponding author. The data are not publicly available due to privacy or ethical restrictions.
